# Pulsatile Trabecular Meshwork Motion: An Indicator of Intraocular Pressure Control in Primary Open-Angle Glaucoma

**DOI:** 10.3390/jcm11102696

**Published:** 2022-05-10

**Authors:** Rong Du, Chen Xin, Jingjiang Xu, Jianping Hu, Huaizhou Wang, Ningli Wang, Murray Johnstone

**Affiliations:** 1 Beijing Tongren Eye Center, Beijing Institute of Ophthalmology, Beijing Tongren Hospital, Capital Medical University, Beijing 100730, China; drdurong@ccmu.edu.cn (R.D.); hjp@mail.ccmu.edu.cn (J.H.); whz@ccmu.edu.cn (H.W.); wningli@ccmu.edu.cn (N.W.); 2 School of Physics and Optoelectronic Engineering, Foshan University, Foshan 528000, China; jjxu@uw.edu; 3 Department of Ophthalmology, University of Washington, Seattle, WA 98195, USA; murrayj2@uw.edu

**Keywords:** trabecular meshwork, phase-sensitive optical coherent tomography, pulsatile motion, IOP fluctuation, primary open-angle glaucoma

## Abstract

(1) Background: To investigate the value of pulsatile trabecular meshwork (TM) motion in predicting the diurnal intraocular pressure (IOP) fluctuation of primary open-angle glaucoma (POAG). (2) Methods: This cross-sectional study recruited 20 normal patients and 30 patients with POAG. Of the POAG group, 20 had stable diurnal IOP and 10 had high IOP fluctuation. A clinical prototype phase-sensitive optical coherence tomography (PhS-OCT) model was used to measure TM pulsatile motion with maximum velocity (MV) and cumulative displacement (CDisp). (3) Results: MV and CDisp were higher in the external region in both normal and POAG patients. All MV and CDisp reduced significantly in the POAG group (*p* < 0.001). In the POAG group, except MV in the external region (*p* = 0.085), MV and CDisp in the nasal area were significantly higher than those in the temporal area (*p* < 0.05). The MV and CDisp in the external region in the nasal area of POAG patients with high IOP fluctuation were much lower than those with stable IOP (*p*_EMV3_ = 0.031, *p*_ECDisp3_ < 0.001); (4) Conclusions: Pulsatile TM motion reduced in POAG patients relevant to the level of diurnal IOP fluctuation. This study presents the segmental variance of TM stiffness in human living eyes and suggests the clinical potential of the measurement of pulsatile TM motion with PhS-OCT for the evaluation of diurnal IOP fluctuation.

## 1. Introduction

Glaucoma is a leading cause of irreversible blindness around the world [1]. Primary open-angle glaucoma (POAG) is the most common type, with characteristics of optic neuropathy and visual field defects. Intraocular pressure (IOP) elevation is widely accepted as a major cause of POAG, and IOP control is the most reliable treatment [2,3]. Although IOP has become widely accepted as a major cause of POAG, how IOP is regulated and why it increases remain an enigma.

IOP measurement is the approach most frequently used to evaluate POAG treatment adequacy. Large and irregular IOP fluctuations may the cause loading and unloading of stress; the tissue is unable to compensate, and then damage occurs. Studies have proven that IOP is highly variable, and large IOP fluctuations are an independent risk factor for POAG development and progression [4,5]. Thus, 24 h IOP monitoring is recommended [6].

Because the measurement of IOP for 24 h is inconvenient and time-consuming [7], it would be valuable if we could estimate the fluctuation of IOP by less complex means. IOP homeostasis depends on the normal function of the aqueous outflow system, especially the trabecular meshwork (TM) outflow pathway [8]. Many experimental and clinical studies provide evidence that bulk aqueous humor outflow is characteristically pulse-dependent [9]. The pressure-sensitive bulk motion of the TM changes the dimensions of Schlemm’s canal (SC), which functions as a compressible chamber and is a prerequisite for the pulsatile flow pattern [10].

Multiple lines of evidence document the progressive decrease and eventual loss of pulsatile flow in glaucoma patients as the disease progresses [11]. The elastic property of TM provides a grounding in the pumping of aqueoushumor. The TM pulsatile motion represents TM deformability to the cardiac-induced ocular pulse amplitude. As an important regulatory site, TM dysfunction plays a critical role in the pathogenesis of POAG [12,13]. TM stiffness, defined as the tissue’s resistance to deform, is one of the essential factors that affects the ability to induce the pulsatile motion [14].

Phase-sensitive optical coherence tomography (PhS-OCT) has been found to quantify the pulsatile TM motion in vivo with high repeatability and reliability [15]. The quantified pulsatile motion is thought to be representative of TM stiffness. Recently, one study identified a reduced pulsatile TM motion in patients with PAOG by using PhS-OCT [16]. However, how effectively the pulsatile movement of TM measured by PhS-OCT reflects IOP fluctuation has not yet been evaluated. Here, we report a cross-sectional study designed to investigate the relationship between the pulsatile movement of TM and IOP fluctuation in normal and POAG subjects.

## 2. Materials and Methods

### 2.1. Subjects

This cross-sectional study was conducted at the Beijing Tongren Hospital, Capital Medical University, between August and December 2020. The study was approved by the Ethical Review Committee of Beijing Tongren Hospital (TRECKY2018-066). The study adhered to the tenets of the Declaration of Helsinki, and each subject signed an informed consent document.

All the recruited subjects received a comprehensive ocular examination of the right eye, including a review of their medical history, best-corrected visual acuity, refraction, a slit lamp, and a stereoscopic optic disc examination. Blood pressure (BP) was measured using an automatic BP device (OMRON Heem-907 blood pressure monitor, OMRON, Kyoto, Japan). IOP was measured by using Goldmann applanation tonometry (GAT). We used an IOL-Master 700 (Carl Zeiss Meditec AG, Jena, Germany) to measure the axial length (AL), central corneal thickness (CCT), and anterior chamber depth (ACD). We admitted POAG subjects to the hospital to provide 24 h IOP monitoring. We measured IOP in the seated position at the following seven times of day using GAT: 2:00 AM, 6:00 AM, 8:00 AM, 10:00 AM, 2:00 PM, 6:00 PM, and 10:00 PM. IOP fluctuation was defined as the difference between the maximum IOP and the minimum IOP in a day.

Normal subjects had no other ocular disease except dry eye or myopia (0~−3.0 Diopters). POAG was defined as the presence of a normal and open-angle gonioscopy, untreated IOP without medication > 21 mmHg, glaucomatous visual field loss confirmed on the subsequent visual field test, and corresponding glaucomatous optic nerve damage evidenced by stereoscopic fundus examination and the images of an optical coherence tomograph. All POAG patients were treated with topical medication for at least three months (18.6 ± 3.4 weeks). For these POAG patients, IOP was measured twice a week after medications were administered. The prescription for POAG was as follows: 1. Prostaglandins; 2. β-blocker; 3. Brinzolamide. All the recorded IOP values measured in the clinic during office hours were less than 21 mmHg while on medication. The exclusion criteria were as follows: (1) previous ocular trauma or surgery; (2) ocular diseases other than POAG; (3) high myopia with aspherical equivalent worse than −3.0 Diopters; (4) known diabetes or cardiac disease.

### 2.2. PhS-OCT Examination and Data Processing

**Figure 1:**
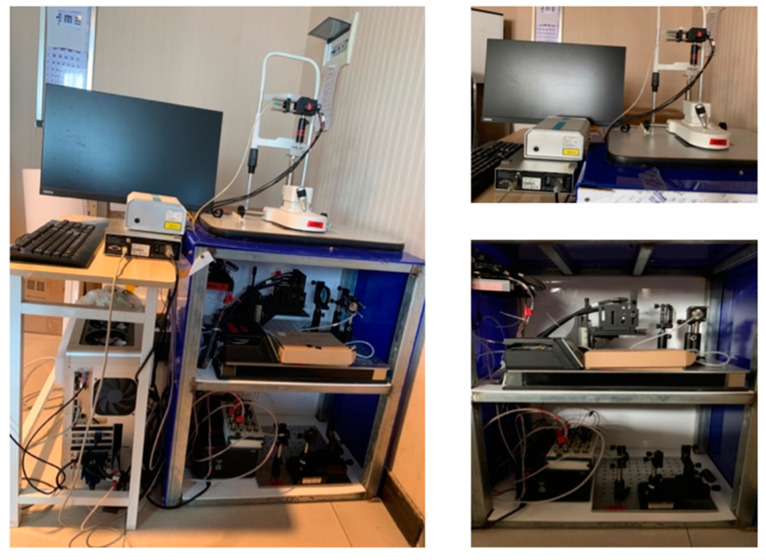
Photos of the clinic’s phenotype of phase-sensitive optical coherence tomography.

The clinical PhS-OCT prototype (Figure 1) is composed of three parts: (1) a spectral-domain OCT system with the light source of a wavelength of 1310 nm with a spectral bandwidth of 100 nm; (2) a digital pulsimeter (Powerlab, ML 866, Colorado Springs, CO, USA) for cardiac signal recording; (3) an external controlling unit for synchronizing the OCT and cardiac signals. The theoretical axial resolution was ~5.5 µm, and the lateral resolution was ~16 µm in tissue. The subject was seated facing straight ahead in a slit-lamp style chin rest with a headrest support, and a digital pulsimeter was placed on the tip of the index finger. The subjects followed an external fixation target with their eyes without moving their head during PhS-OCT imaging. We scanned the temporal and nasal 3.5 mm limbal region of the TM. Each scan lasted for 5 s, creating a dataset containing 2000 OCT B-scans (400 B-scans/s).

**Figure 2:**
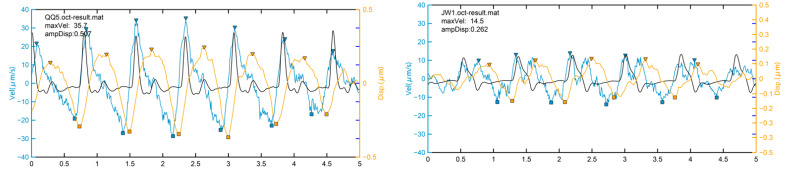
Representative of trabecular motion (TM) synchronized with heartbeat. The TM motion is synchronized with the heartbeat. The black curves represent the heartbeat. The blue curves indicate the instantaneous velocity of the TM in each cycle, while the orange lines demonstrate the cumulative displacement of the TM, which moves away from the original location at each check point. The inverted triangle indicates the peak value of the traced curve, while the square presents the valley value. The positive of the velocity or the displacement represents the TM moving outwards to the sclera in the systole; the negative velocities represent the TM moving inwards towards the anterior chamber during the diastole. The maximum velocity means the maximum instantaneous velocity during a cycle. The figures show two representatives of the TM motion in normal (**left**) and POAG patients (**right**).

For each dataset, the velocity waveform of each pixel was used to generate a velocity waveform tracing. A proprietary technique was used to compensate for the bulk involuntary motion occurring during the measurement. Between adjacent B-scans, we analyzed the phase shift of each pixel in the OCT signals, then calculated instantaneous velocity based on the difference between the two B-scan images. A mask derived from the cardiac pulse and harmonic frequency filtered the motion waveforms. We selected two regions of interest for each scan. The internal region of TM was defined as one-third of the distance anterior to the sclera spur along the line between Schwalbe’s line and the sclera spur. The external region of the TM was selected as the area next to the SC lumen. The maximum velocity (MV) and cumulative displacement (CDisp) were then calculated (Figure 2), providing first an internal TM maximum velocity (IMV) and displacement (ICDisp), then an external TM Velocity (EMV) and displacement (ECDisp). For details on how to characterize the Phs-OCT system for dynamic displacement measurement, please refer to our previous studies [15,17].

All the examinations were performed by Dr. XC, who was masked to the diagnosis of the subjects. PhS-OCT imaging was performed at 10:00 AM, with the temporal and nasal limbal region scanned in each subject. Three successive datasets were collected in an examination on one day to assess the repeatability of the imaging. The scans were repeated two weeks later to determine the reliability of the PhS-OCT technique on separate exam days.

### 2.3. Statistical Analysis

Descriptive statistics are presented as the mean ± standard deviation (SD). The significant differences of the parameters of the pulsatile TM motion between normal patients and patients with POAG were determined using an unpaired *t*-test based on the analysis of normality (Shapiro–Wilk test). The intraclass correlation coefficient (ICC) was used to assess the repeatability and reproducibility of the pulsatile TM motion measurements in the PhS-OCT images. Each comparison was labeled as significant when *p* < 0.05.

## 3. Results

### 3.1. Demographic and Baseline Characteristics of the Subjects

Twenty normal and 30 POAG subjects were recruited in this study. The mean ages of the normal and POAG groups were 41.3 ± 9.3 years and 44.6 ± 9.5 years (*p* = 0.992), respectively. The IOP at 10:00 AM was not significantly different between groups (*p* = 0.111): 15.4 ± 1.6 mm Hg in the normal group and 15.3 ± 2.0 mmHg in the POAG group. The baseline characteristics of all participants are shown in Table 1 and Table 2.

### 3.2. Repeatability and Reliability

The ICCs of IMV, EMV, ICDisp, and ECDisp for the three continuous scans in the same region were 0.953, 0.937, 0.917, and 0.914, respectively. The ICCs of IMV, EMV, ICDisp, and ECDisp for the two images captured on separate days were 0.973, 0.884, 0.913, and 0.782, respectively.

### 3.3. Difference in MV and CDisp between Healthy and POAG Eyes

**Figure 3:**
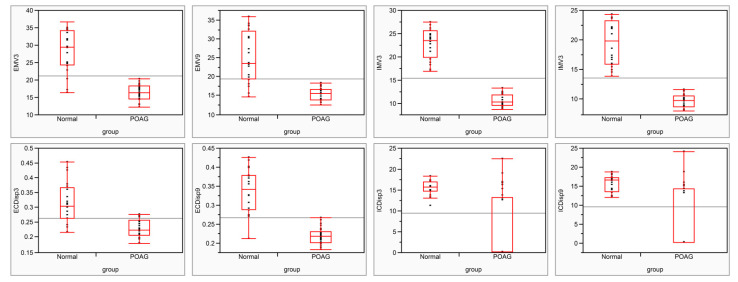
The pulsatile trabecular meshwork (TM) motion in normal and POAG eyes. MV—maximum velocity; CDisp—cumulative displacement; EMV3—MV in the external region of TM in the nasal area; EMV9—MV in the external region of TM in the temporal area; IMV3—MV in the internal region of TM in the nasal area; IMV9—MV in the internal region of TM in the nasal area; ECDisp3—CDisp in the external region of TM in the nasal area; ECDisp9—CDisp in the external region of TM in the temporal area; ICDisp3—CDisp in the external region of TM in the nasal area; ICDisp9—CDisp in the external region of TM in the temporal area. POAG—primary open-angle glaucoma.

The results for pulsatile TM motion in all the recruited eyes are presented in Figure 3. In both the nasal and temporal areas, MV and CDisp in the internal and external regions were significantly lower in POAG eyes than in normal eyes (*p* < 0.001).

In normal eyes, in both the internal and external regions, the MV in the nasal area was significantly higher than that in the temporal area. The CDisp in the external region of the TM in the nasal area was much higher than that in the temporal area. However, the CDisp in the internal region of the TM in the nasal area was similar to the temporal area (Table 2). Similar to normal subjects, the MVs in the nasal area were significantly higher than those in the temporal area of the eyes with glaucoma. However, in glaucoma eyes, the CDisp in both the external and internal regions of the TM in the nasal area was much higher than that in the temporal area (Table 3).

Using the diurnal IOP amplitude (IOP_highest_–IOP_lowest_) as a categorical factor, the glaucoma eyes could be divided into two groups. Twenty eyes had stable IOP, defined as a diurnal IOP amplitude of ≤8 mm Hg, and ten eyes had fluctuating IOP (>8 mmHg). The mean IOP amplitudes were 4.0 ± 1.5 mmHg and 9.9 ± 2.2 mm Hg in the stable and fluctuating groups, respectively. The results showed that, compared with normal subjects, all the parameters of TM pulsatile motion decreased dramatically in both the stable and fluctuating groups (*p* < 0.01) in those with glaucoma. Moreover, the EMV3 (15.1 ± 2.3 µm/s) and ECDisp3 (0.205 ± 0.021 µm) in the fluctuating group were significantly lower than those in the stable group (19.9 ± 2.0 µm/s, pEMV3 = 0.031; 0.237 ± 0.030 µm, pECDisp3 < 0.001).

## 4. Discussion

In this study, we firstly demonstrated the good reliability and repeatability of TM motion quantification, as previously reported [15]. PhS-OCT was used to characterize the TM motion change with accommodation [15]. Our results showed that pulsatile TM motion was reduced in glaucoma compared with normal subjects. The pulsatile motion of TM originates from the ocular pulse caused by the oscillatory change in choroidal volume during the cardiac cycle [9]. Pulsatile TM motion reflects TM stiffness, which becomes abnormal in glaucoma [14,16]. Previously, the PhS-OCT system has been shown to be able to differentiate POAG from healthy subjects [17]. The study found that parameters of pulse-dependent TM motion were better able to predict the presence of glaucoma than measurements of outflow facility, or IOP measurements during clinic hours [17].

In this study, we matched the normal and POAG patients in terms of age, heart rate, and mean arterial pressure, which potentially correlate with TM motion. Although the IOP was well controlled with topical medication in the POAG patients, the TM motion was dramatically reduced nasally and temporally in both the internal and external TM regions. Recently, Li G et al. reported that, in corticosteroid-treated mice, SC was more resistant to collapse in elevated IOPs. The study, performed by estimates using inverse finite element modeling, was consistent with increased TM stiffness [18]. Additionally, Wang K et al. estimated human TM stiffness by numerical modeling and found that normal TM stiffness was lower than in glaucoma patients [16].

We also found that the pulsatile TM motion presented differently in different segments. Motion the in nasal region was much stronger than that in temporal area in both normal and glaucomatous eyes. However, we did not identify a regional progression difference of TM regions in glaucoma. Studies have documented that aqueous outflow is not homogeneous, but segmental [19,20]. Previous studies have reported that aqueous fluid is predominantly drained in the nasal and inferior quadrants [19]. The segmental labeling of the TM in multiple studies has provided evidence of regions of preferential aqueous outflow in both normal and glaucomatous eyes [21]. The circumference of the TM can be divided into regions of high, medium, and low flow, based on angiographic imaging or the distribution of fluorescent microspheres [22]. Moreover, a study showed that TM stiffness in high-flow wedges was softer than that in low-flow wedges for both normal and glaucomatous eyes [22].

Many studies have highlighted the importance of stable IOP fluctuations for preventing the progression of POAG. We found that the TM motion in the external region, next to SC, decreased dramatically in POAG eyes with high IOP variation during 24 h monitoring, compared to those with stable diurnal IOP. This high variance of 24 h IOP fluctuation reflects a loss of normal IOP homeostasis, and suggests the malfunctioning of the aqueous outflow system. The IOP peak typically occurs at night, reportedly related to the supine position and changes in ocular pulsations during sleep. The diurnal IOP fluctuation present in glaucoma, especially the IOP peak, correlates with the progression of visual field defects.

The TM plays a vital role in maintaining IOP homeostasis, reflecting the tissue’s importance in preserving biomaterial properties, such as stiffness. It has been reported that pulsatile fluid in the aqueous vein was induced by an IOP increase after a water drinking test [11], which suggests that TM pumping functions in reaction to IOP variation. Recent studies report that SC shear stress and TM strain may act together as mechanosensory factors providing the homeostatic regulation of aqueous outflow and IOP [23]. A feed-forward loop involving alterations in TM stiffness may exacerbate malfunction of TM cells with further aberrations of the extracellular matrix of TM beams, the spaces between beams, and the juxtacanalicular tissue. Our study indicates that reduced TM motion, reflecting increased stiffness, may be relevant to the abnormal IOP homeostasis of glaucoma. Moreover, the results showed that the movement of the TM in the external region in glaucoma eyes with high IOP fluctuation decreased significantly. The external region of TM measured in this study mainly covered the juxtacanalicular TM region. Decreased movement indicates the TM becoming stiffer. This leads to the inability of TM to react to IOP transience, disturbing IOP homeostasis. The relationship between worse pulsatile TM motion and higher diurnal IOP fluctuation implicates the clinical potential of PhS-OCT for the differentiation of POAG and the efficient evaluation of POAG treatment.

There are several limitations to this study. (1) We have presented the segmental difference of TM pulsatile motion in normal and glaucoma subjects. Whether TM pulsatile motion represents regions that are especially vulnerable to damage requires further study. (2) The glaucoma subjects recruited in this study were relatively young, and they were treated with topical medications which might have altered the biomechanical properties of TM and affected aqueous outflow and IOP in currently unknown ways. Further studies are needed to compare normal tension glaucoma and healthy controls treated with the same medication.

## 5. Conclusions

Our study found segmental TM differences, with the nasal and inferior areas experiencing the greatest motion in both normal and glaucomatous eyes. The pulsatile motion was greater in the external than the internal portion of the TM. Pulsatile TM motion was decreased in glaucoma eyes compared with normal eyes, and imaging was able to detect glaucoma patients with large diurnal fluctuations. Imaging pulsatile TM motion may provide valuable insight into the pathophysiology of the aqueous outflow system in glaucoma. Imaging TM motion abnormalities may also help identify those at risk for fluctuations in IOP that are missed by measurements during clinic hours.

## Figures and Tables

**Table 1:** Demographic of subjects.

	Normal	POAG	*p*-Value
Age (years)	41.3 ± 9.3	44.6 ± 9.5	0.992
Sex (F:M)	11/9	10/20	0.154
Axial length (mm)	24.04 ± 0.43	24.03 ± 0.28	0.069
Central corneal thickness (µm)	527 ± 28	540 ± 33	0.342
Heart rate	70.3 ± 8.8	72.9 ± 9.4	0.406
Mean arterial pressure (mmHg)	87.9 ± 7.5	90.4 ± 6.2	0.445
IOP (mmHg)	15.4 ± 1.6	15.3 ± 2.0	0.111
Mean deviation (dB)		−9.32 ± 1.58	

POAG—primary open-angle glaucoma; F:M—Female: Male; IOP—intraocular pressure.

**Table 2:** Demographic of POAG subjects.

	IOP Stable	IOP Fluctuant	*p*-Value
Age (years)	44.6 ± 14.5	46.4 ± 15.2	0.630
Sex (F:M)	8/12	4/6	0.833
Follow-up (weeks)	20.8 ± 4.2	19.7 ± 2.9	0.115
Axial length (mm)	24.34 ± 0.50	24.42 ± 0.50	0.982
Central corneal thickness (µm)	544 ± 37	537 ± 28	0.287
Heart rate	72.1 ± 4.6	73.5 ± 5.1	0.842
Mean arterial pressure (mmHg)	90.8 ± 9.5	89.4 ± 11.5	0.333
IOP (mmHg)	16.1 ± 3.1	16.2 ± 2.5	0.471
Mean deviation (dB)	−11.03 ± 3.09	−13.15 ± 4.01	0.127

**Table 3:** The pulsatile TM motion in normal and POAG eyes.

	Nasal	Temporal	*p*-Value
Normal			
EMV, µm/s	28.5 ± 6.3	25.2 ± 6.8	0.002
IMV, µm/s	22.8 ± 3.2	19.5 ± 3.7	<0.001
ECDisp, µm	0.341 ± 0.063	0.305 ± 0.064	0.036
ICDisp, µm	0.271 ± 0.063	0.248 ± 0.064	0.253
POAG			
EMV, µm/s	16.3 ± 2.2	15.3 ± 1.6	0.085
IMV, µm/s	11.2 ± 1.9	9.7 ± 1.2	0.01
ECDisp, µm	0.231 ± 0.031	0.218 ± 0.021	0.037
ICDisp, µm	0.207 ± 0.038	0.156 ± 0.034	<0.001

TM—trabecular meshwork; POAG—primary open-angle glaucoma; MV—maximum velocity; CDisp—cumulative displacement; EMV—MV in the external region of TM; IMV—MV in the internal region of TM; ECDisp—CDisp in the external region of TM; ICDisp—CDisp in the internal region of TM.

## Data Availability

The data presented in this study are available from the corresponding author upon reasonable request.
